# Metabolome Profiling in the Plasma of Dogs with Idiopathic Dilated Cardiomyopathy: A Multiplatform Mass-Spectrometry-Based Approach

**DOI:** 10.3390/ijms242015182

**Published:** 2023-10-14

**Authors:** Ivana Rubić, Stefan Weidt, Richard Burchmore, Alan Kovačević, Josipa Kuleš, Peter David Eckersall, Marin Torti, Ines Jović, Mislav Kovačić, Jelena Gotić, Renata Barić Rafaj, Predrag Novak, Marko Samardžija, Vladimir Mrljak

**Affiliations:** 1 Laboratory of Proteomics, Clinic for Internal Diseases, Faculty of Veterinary Medicine, University of Zagreb, 10000 Zagreb, Croatia; irubic@vef.hr; 2 Glasgow Polyomics, Wolfson Wohl Cancer Research Centre, University of Glasgow, Glasgow G61 1QH, UK; stefan.weidt@glasgow.ac.uk (S.W.); richard.burchmore@glasgow.ac.uk (R.B.); 3 Department of Clinical Veterinary Medicine, Vetsuisse Faculty, University of Bern, 3012 Bern, Switzerland; alan.kovacevic@unibe.ch; 4 Department of Chemistry and Biochemistry, Faculty of Veterinary Medicine, University of Zagreb, 10000 Zagreb, Croatia; jkules@vef.hr (J.K.); rrafaj@vef.unizg.hr (R.B.R.); 5 Institute of Biodiversity, One Health and Veterinary Medicine, College of Medical, Veterinary and Life Sciences, University of Glasgow, Glasgow G61 1QH, UK; david.eckersall@glasgow.ac.uk; 6 Interdisciplinary Laboratory of Clinical Analysis of the University of Murcia (Interlab-UMU), Department of Animal Medicine and Surgery, Veterinary School, University of Murcia, 30100 Murcia, Spain; 7 Clinic for Internal Diseases, Faculty of Veterinary Medicine, University of Zagreb, 10000 Zagreb, Croatia; mtorti@vef.hr (M.T.); ijovic@vef.hr (I.J.); jselanec@vef.hr (J.G.); 8 Department of Biology, University of Osijek, 31000 Osijek, Croatia; k.mislav@gmail.com; 9 Department of Chemistry, Faculty of Science, University of Zagreb, 10000 Zagreb, Croatia; pnovak@chem.pmf.hr; 10 Reproduction and Obstetrics, Faculty of Veterinary Medicine, University of Zagreb, 10000 Zagreb, Croatia; smarko@vef.unizg.hr

**Keywords:** idiopathic dilated cardiomyopathy (iDCM), plasma samples, dogs, metabolomics, chromatography, mass spectrometry

## Abstract

Dilated cardiomyopathy is one of the important diseases in dogs and humans. The second most common cause of heart failure in dogs is idiopathic dilated cardiomyopathy (iDCM), which results in heart failure or sudden cardiac death due to arrhythmia. This study aimed to determine changes in the plasma metabolome of dogs with iDCM compared to healthy dogs. For that purpose, a multiplatform mass-spectrometry-based approach was used. In this study, we included two groups of dogs: 12 dogs with iDCM and 8 healthy dogs. A total of 272 metabolites were detected in the plasma samples of dogs by combining three approaches but four MS-based platforms (GC-MS, LC-MS (untargeted), LC-MS (targeted), and FIA-MS (targeted) methods). Our findings demonstrated changes in the canine plasma metabolome involved in the development of iDCM, including the different concentrations of amino acids, biogenic amines, acylcarnitines, triglycerides and diglycerides, sphingomyelins, and organic acids. The results of this study will enable the detection and monitoring of pathophysiological mechanisms involved in the development of iDCM in the future.

## 1. Introduction

Idiopathic dilated cardiomyopathy (iDCM), a primary myocardial disease, is the second most common acquired heart disease in dogs, which results in congestive heart failure or sudden cardiac death due to arrhythmias. It is characterised by systolic dysfunction and eccentric hypertrophy of the left ventricle, and in some cases also of the right ventricle [1].

The exact pathomechanisms that leads to the development of iDCM have yet to be fully clarified, and the cause remains unknown. iDCM is a genetic disease, with a slow onset, and is typically detected in middle-aged or older dogs. Male dogs appear to be more affected than female dogs [2]. It is also found in humans, mainly affecting young and adults, and can result in heart transplantation at a younger age [3]. Therefore, the discovery of iDCM at an early stage (asymptomatic or occult stage) and the timely initiation of therapy to slow the progression of the disease is extremely important and poses a significant challenge for veterinarians worldwide [4,5].

The clinical progression of iDCM can be described as occurring in three distinct stages: stage I—characterised by damage on the cellular or genetic level, stage II—characterised by cardiac remodelling, and stage III—characterised by presence of clinical signs of congestive heart failure. Whilst the first two stages can persist for years, stage III has a short duration of several months [6]. The most common clinical signs of iDCM are those of congestive heart failure and include respiratory distress due to pulmonary oedema or pleural effusion, exercise intolerance and general weakness, ascites, and weight loss. In dogs with arrhythmias, syncope and sudden cardiac death due to malignant arrhythmias are common [7].

The prognosis of the disease is generally unfavourable. The average survival time in dogs with overt or clinical iDCM is six to nine months. Namely, about 50% of dogs with the symptomatic form of iDCM die within three months, while 20% survive for two years.

Possible causes of altered cardiac functions in patients with iDCM may be related to changes in cardiac muscle metabolite levels and metabolic pathway disorders involved in the development of the iDCM. The metabolites involved in heart disease can be discovered using metabolomics and metabolomics approaches to investigate the molecular processes responsible for the development of heart disease. It can also provide valuable information that will help identify early forms of cardiovascular diseases [8,9].

Numerous studies in human medicine have used metabolomics methods to detect small molecules that would serve as biomarkers for cardiovascular diseases. Thus, Shah et al. (2012) generated a metabolomics profile of patients with risk of coronary artery disease [10]. Wang et al. (2011) performed a metabolomics analysis to investigate metabolites as markers for predicting cardiovascular disease [11]. Diaz et al. (2022) used the GC-MS system to investigate the metabolomics profile of 52 patients with systolic heart failure [12].

Recent metabolomics studies have also been performed to investigate dilated cardiomyopathy. Alexander et al. (2011) performed a metabolomics analysis in plasma of 39 patients diagnosed with dilated cardiomyopathy and identified 451 metabolites [13]. West et al. (2016) quantified more than 130 metabolites investigated in mice with dilated cardiomyopathy by targeted metabolomics analysis and discovered metabolomics pathways relevant to cardiac metabolism [14]. They noted a decreased energy metabolism in the heart with dilatation disorder and stronger degeneration of myofibrils and collagen. Using an untargeted metabolomics approach, Zhao et al. (2020) generated the plasma metabolomics profile of patients with dilated cardiomyopathy [15]. They suggested that the metabolism of glycerophospholipid and α-linolenic acid was significantly altered. Biopterin, *O*-acetyl-L-carnitine, and sphingomyelin were shown to be the most abundant metabolites contributing to the difference between patients with dilated cardiomyopathy and healthy controls [15].

The aim of this study was to investigate metabolite profiles and possible changes in the plasma metabolome in dogs with iDCM using liquid and gas chromatography coupled to mass spectrometry, as well as flow-injection analysis, with our ultimate goal being to suggest new biomarkers of iDCM in the canine species. Additionally, identifying unique metabolic signatures and differences across different disease manifestations would be a useful tool to better understand the pathophysiology of iDCM.

## 2. Results

### 2.1. The Metabolomics Data Set in Dogs with Diagnosed iDCM

**Figure 1:**
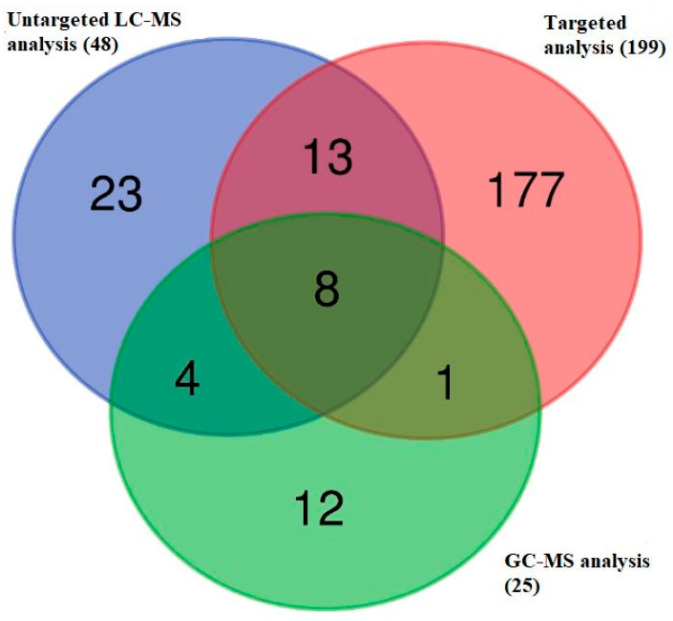
Metabolites identified in plasma of dogs with iDCM and healthy dogs using a Venn diagram. The untargeted liquid chromatography coupled to mass spectrometry (LC-MS) metabolomics approach identified 48 metabolites, the targeted metabolomics approach (Biocrates analysis) identified 199 metabolites, and 25 metabolites were identified by the gas chromatography coupled to mass spectrometry (GC-MS) metabolomics approach. A total of 272 metabolites were identified using three approaches.

A total of 272 different metabolites were identified using an untargeted LC-MS and targeted (combining LC-MS and FIA-MS) metabolomics analysis and a GC-MS metabolomics assay in a plasma sample of dogs with iDCM (Figure 1, Table S1). The Venn diagram indicates the identification of 26 metabolites using more than one platform. Among them, 13 metabolites were detected by untargeted LC-MS, targeted assay, and GC-MS methods; 9 metabolites were measured by the targeted metabolomics approach (Biocrates Kit (Biocrates Life Science AG, Innsbruck, Austria)) and the GC-MS method; and 21 metabolites were measured using untargeted LC-MS and targeted approach. A total of eight metabolites were measured using three approaches, but four MS-based platforms.

### 2.2. Untargeted Metabolomics Approach

The metabolomics analysis resulted in detecting 2636 features in plasma samples of 8 dogs with iDCM and 12 healthy dogs using an untargeted metabolomics approach on the LC-MS platform (Table S2A). Among them, a total of 48 metabolites were identified on the basis of the mass/retention time matched to known standards in the Polyomics integrated Metabolomics Pipeline (PiMP) software available at http://polyomics.mvls.gla.ac.uk (accessed on 15 July 2023). According to the statistical *p*-value (FDR) of <0.05, a total of 394 features were significantly changed (Table S2B). Among them, a total of 10 metabolites were identified by reference to authentic standards (Table 1).

In addition, cystine and 4-hydroxyproline were lower in abundance, while creatinine, 3-hydroxybutanoate, orotate, lactate, carnitine, *cis*-aconitate, *O*-acetyl-L-carnitine, and 3-methylhistidine were higher in abundance in dogs with iDCM (Table 1).

### 2.3. Targeted Metabolomics Approach

Targeted metabolomics analysis resulted in a total of 199 metabolites (from a total possible of 408, from the Absolute IDQ p400 kit (Biocrates Life Sciences AG, Innsbruck, Austria)) in plasma samples of 20 dogs, which were used for further statistical analysis (Table S3A). The datasets of 199 metabolites were classified into several groups. Among them, 19 metabolites belonged to the group of amino acids, 9 metabolites were biogenic amines, 1 metabolite was a monosaccharide, 4 metabolites were acylcarnitines, 9 metabolites belonged to the group of cholesteryl esters, 8 metabolites belonged to the group of diglycerides, and 29 metabolites belonged to the group of triglycerides. A total of 11 metabolites were lysophosphatidylcholines, 87 were phosphatidylcholines, and 22 metabolites were sphingomyelins.

Metabolites were quantified according to the manufacturer’s guidelines using the Biocrates MetIDQ^TM^ software Version Boron (Biocrates Life Sciences AG, Innsbruck, Austria) for targeted metabolomics data processing and management. In total, 31 were fully validated as absolutely quantitative, 166 as relatively quantified, and 2 metabolites were quantified with restrictions.

The univariate metabolomics analysis identified eight metabolites with significantly different concentrations between two experimental groups by LC-MS and FIA-MS analysis. Among them, only asymmetric dimethylarginine (ADMA) was identified metabolites by LC-MS analysis, which was significantly higher in dogs with iDCM (*p*-value (FDR): 0.003, log2 (FC): 0.91).

By the FIA-MS approach, we detected seven significant metabolites divided into groups of triglycerides (3), diglycerides (3), and sphingomyelin (1) with different abundance between dogs with iDCM and healthy dogs (Table S3B). All of the metabolites with lower levels were different triglycerides (TG). Sphingomyelin (SM (43:1)), diacylglycerol 36:3 (DG (36:3)), diacylglycerol 36:2 (DG (36:2)), and DG (34:1) were significantly higher (Table S3B).

### 2.4. GC-MS-Based Metabolomics

The GC-MS metabolomics analysis identified 25 metabolites in plasma samples of 20 dogs. Among them, a total of 10 were amino acids, 8 organic acids, 3 carbohydrates, 3 fatty acids, and 1 was a sugar alcohol (Table S4A). According to the *p*-value (FDR), none of the detected metabolites were statistically significant.

### 2.5. Identification of Metabolites Showing Differential Abundance in the Plasma Metabolome of Dogs with iDCM and Healthy Dogs

**Figure 2:**
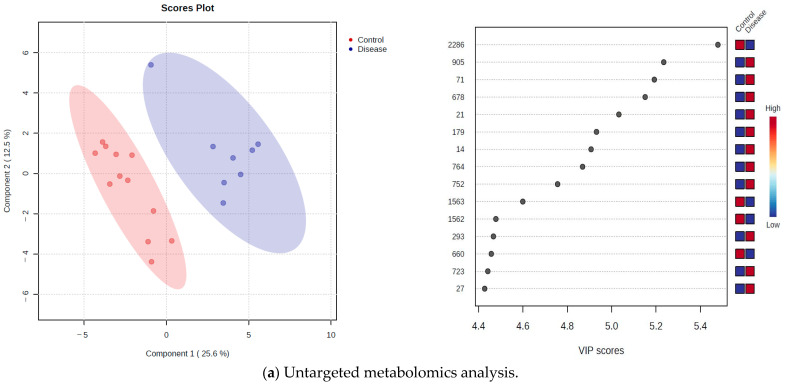

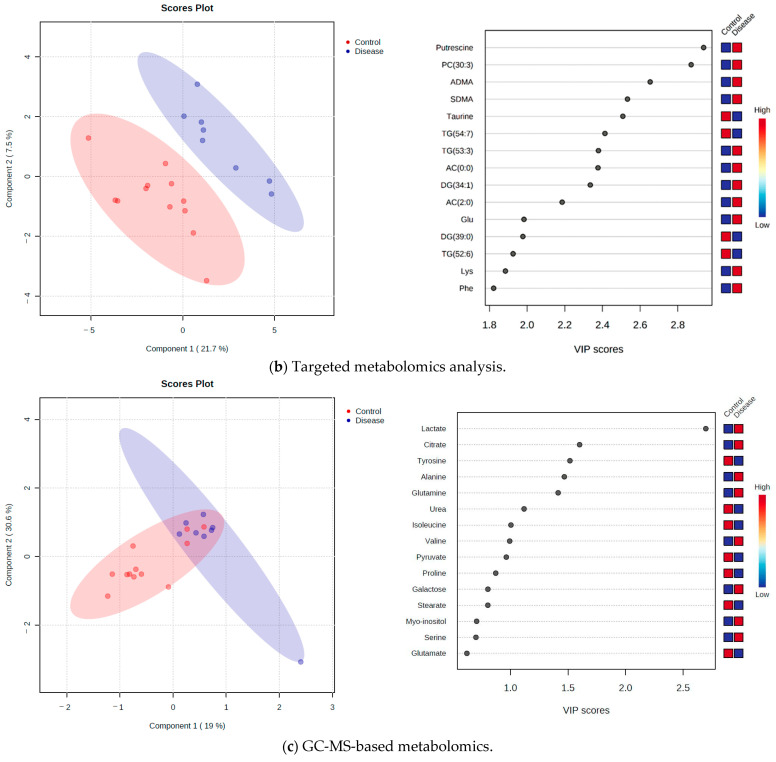
Partial least squares discriminant analysis (PLS-DA) score plots were performed for the 8 analysed plasma samples of dogs with iDCM and 12 healthy dogs by untargeted LC-MS metabolomics analysis (**a**), targeted metabolomics analysis (**b**), and GC-MS-based metabolomics (**c**) (left panels). Variable importance in projection (VIP) scores for the 15 most influential metabolites identified by PLS-DA analysis in the untargeted LC-MS metabolomics (**a**), targeted metabolomics (**b**), and GC-MS metabolomics analysis (**c**) (right panels). The intensity of the coloured boxes on the right represents the relative intensities/concentrations of the corresponding metabolite in two group of dogs (healthy, dogs with iDCM).

A partial least squares discriminant analysis (PLS-DA) enabled the identification of metabolites that were the most discriminating between dogs with iDCM and healthy dogs. This analysis represented a clear intergroup separation between the two experimental groups of dogs investigated by untargeted LC-MS (Figure 2a), targeted metabolomics (Figure 2b), and GC-MS-based metabolomics (Figure 2c). The validity of PLS-DA was confirmed by cross-validation and indicated that the best classifier model comprised two components for untargeted LC-MS metabolomics (R2 = 0.91, Q2 = 0.64), five components for targeted metabolomics (R2 = 0.99, Q2 = 0.10), and five components for GC-MS metabolomics (R2 = 0.85, Q2 = −3.27).

The overall variable importance in the projection (VIP) score of the PLS-DA produced the list of the 15 most influential metabolites/features contributing to the separation in the PLS-DA plot. The coloured boxes on the right indicate the relative intensities of the corresponding metabolites in each studied group for the untargeted LC-MS metabolomics (Figure 2a) and the relative concentrations for the targeted (Figure 2b) and GC-MS metabolomics (Figure 2c). For the untargeted LC-MS metabolomics analysis, the results showed that the highest VIP score belongs to the peak 2286 (mass: 353.012, RT(s): 750.78) with lower concentration in dogs with iDCM. Metabolite *O*-acetyl-L-carnitine (peak 21) and 3-methylhistidine (peak 27) were identified by the untargeted LC-MS metabolomics approach, for which concentrations were higher in dogs with iDCM (Figure 2a). In the targeted metabolomics, the top five of the most influential metabolites obtained by VIP were as follows: putrescine, phosphatidylcholine PC (30:3), asymmetric dimethylarginine (ADMA), symmetric dimethylarginine (SDMA), and taurine. Among them, putrescine had the highest VIP score. The PC (30:3), ADMA, and SDMA were higher in dogs with iDCM in comparison to healthy dogs, while the concentration of taurine was lower (Figure 2b). For the GC-MS-based metabolomics, the results showed that the highest VIP score belonged to the metabolite lactate with higher concentration in dogs with iDCM. Tyrosine was lower in the group of dogs with iDCM in comparison to the healthy group, while the concentrations of citrate, alanine, and glutamine were higher (Figure 2c).

## 3. Discussion

Investigation of metabolites in plasma samples of dogs with iDCM showed that this disease was associated with changes in the plasma metabolomics profile. In this study, we observed the changes in the concentrations of amino acids, organic acids, fatty acids, carbohydrates, sugar alcohols, acylcarnitines, cholesteryl esters, and other types of metabolites.

Plasma samples of dogs with idiopathic dilated cardiomyopathy showed elevated concentrations of lactate detected by an untargeted LC-MS-metabolomics approach. It is thought that lactic acid may be one of the first biomarkers for detection of heart failure [16]. Lactic acid is an important source of energy in the myocardium together with fatty acids and glucose [17]. Recent research suggested that the occurrence of lactic acid in the blood could be a response to stress in heart disease, most often in patients with myocardial ischemia or heart failure [17]. Haas et al. (2021) showed a trend of higher levels in patients with dilated cardiomyopathy using the Biocrates kit (Biocrates Life Sciences AG, Innsbruck, Austria) for targeted metabolomics analysis, but it did not reach significance [18]. Furthermore, elevated concentrations of lactic acid may be associated with reduced expression of the enzyme lactate dehydrogenase A in the blood, but not in myocardial tissue. It is assumed that elevated concentrations of lactic acid in blood can be a consequence of developed systemic diseases such as severe trauma, hypoxemia, and septic shock with poor prognosis [19,20,21].

The investigation by Haas et al. (2021) observed a decreased concentration of 3-hydroxybutyric acid and 3-hydroxybutanoate, respectively, in patients with dilated cardiomyopathy compared to the control group [18]. Interestingly, in dogs with iDCM, a slight increase in the concentration of 3-hydroxybutanoate identified by an untargeted LC-MS-metabolomics approach was observed. The study by Haas et al. (2021) found that the concentrations of metabolites of pyruvate in plasma patients with dilated cardiomyopathy did not differ significantly from the control group [18]. This is in agreement with the results of study performed on dogs with iDCM, in which also no significant change in concentrations of pyruvate was identified by the GC-MS-metabolomics approach.

The metabolite carnitine was identified in the plasma of dogs with iDCM by an untargeted LC-MS metabolomics approach. Carnitine is a key component in the transport of long-chain fatty acids across the inner mitochondrial membrane [22]. It is essential for the oxidation of fatty acids, an important pathway for energy production in the heart [23,24,25]. Carnitine plays an important role in capturing toxic metabolites of long-chain acyl-coenzyme A, which can accumulate in ischemia and cause damage of the sarcolemmal membrane. Deficiency of carnitine prevents the oxidation of fatty acids into carbon dioxide in the mitochondria, which can cause the development of hepatic encephalopathy, hypoketotic hypoglycaemia, or cardiomyopathy [26].

The results obtained in dogs with dilated cardiomyopathy showed elevated concentrations of carnitine identified by untargeted LC-MS-based metabolomics. Research by Pierpont et al. (1989) investigated the elevated concentrations of carnitine in blood of patients with cardiomyopathy [27]. Furthermore, El-Aroussy et al. (2000) compared plasma and urine concentrations of carnitine in patients with congestive heart failure versus the control group [28]. Congestive heart failure occurred as a consequence of cardiomyopathies and rheumatic heart diseases. Interestingly, the results also showed an increased concentrations of carnitine in the plasma of patients with dilated cardiomyopathy and rheumatic heart disease. On the other hand, several clinical studies in patients with dilated cardiomyopathy, acute myocarditis, and rheumatic heart disease have revealed low carnitine concentrations in the left ventricle [29,30,31]. Elevated concentrations of carnitine in plasma and urine may be related with damage of tissue or difficulties in transport of carnitine to cardiac cells [28]. Also, it is considered that elevated concentrations of carnitine in the plasma and urine of patients with congestive heart failure may be marker for myocardial damage and altered left ventricular function [28].

The metabolite *O*-acetyl-L-carnitine, identified using untargeted LC-MS metabolomics approach, displayed an elevated plasma concentrations in dogs with iDCM. Zhao et al. (2020) analysed the plasma of patients with dilated cardiomyopathy and ischemic cardiomyopathy by an untargeted LC-MS-metabolomics approach [15] and found that the concentration of *O*-acetyl-L-carnitine in plasma was elevated in both groups of patients [15]. However, the examination of plasma in healthy patients and patients with hypertension, atrial fibrillation, coronary heart disease, or vascular atherosclerosis suggested elevated levels of *O*-acetyl-L-carnitine, short-chain acyl-carnitine, in all groups of patients with cardiovascular disease compared to healthy patients [32]. Acylcarnitines are thought to be potential diagnostic markers of cardiovascular disease [33]. They can also help in understanding the development of underlying pathological processes and determining cardiometabolic risk in the early stages of cardiovascular disease [34].

Asymmetric dimethylarginine (ADMA) is metabolite identified in the plasma of dogs with iDCM by using the targeted metabolomics approach, and it is considered as a cardiovascular biomarker because of its biological function [35]. ADMA is a modified amino acid and a natural endogenous inhibitor of nitric oxide synthase [36]. It reduces the production of nitric oxide, which can result in endothelial dysfunction and an increased risk of cardiovascular disease. Elevated concentrations of ADMA were found in the plasma of dogs with iDCM compared to healthy dogs. Numerous studies have reported elevated concentrations of ADMA and cardiovascular diseases. Thus, elevated plasma concentrations of ADMA were observed in the plasma of patients with coronary artery disease [37], peripheral arterial disease [38,39], chronic heart failure [40], pulmonary hypertension [41], preeclampsia [42], stroke [43], and hypertrophic cardiomyopathy [44]. Also, the accumulation of ADMA was noticed in the studies of heart valve diseases [45], idiopathic dilated cardiomyopathy [46], congenital heart disease [47], renal failure [48], diabetes [49,50], and atrial fibrillation [51]. Elevated concentrations of ADMA are one of the key indicators of mortality in patients after infarction [52] or coronary heart disease [53]. It is also an independent risk factor for hypertension, coronary artery disease/atherosclerosis, diabetes, and altered renal function that contribute to the development of heart failure [54].

Djordjević et al. (2012) also noted an elevated concentration of ADMA in the plasma of patients with ischemic heart disease [55]. An elevated concentration of ADMA is thought to be indicator of morbidity and mortality in patients with cardiovascular disease [56].

The metabolite 3-methylhistidine was identified in the plasma of dogs by the LC-MS-untargeted metabolomics approach. Elevated concentrations of 3-methylhistidine have been reported in dogs with idiopathic dilated cardiomyopathy compared to healthy dogs and in the plasma of patients with dilated cardiomyopathy compared to healthy patients [13]. Metabolite 3-methylhistidine was considered as potential biomarker for differential diagnosis, progression, and prognosis prediction and treatment monitoring of dilated cardiomyopathy (DCM) [57].

The metabolite orotate was identified in dogs’ plasma by using the untargeted metabolomics approach on the LC-MS platform. Our results noted elevated concentrations in dogs with idiopathic dilated cardiomyopathy. It is shown that orotate can improve the cardiac performance of ischaemic/reperfused rat hearts via the elevation of myocardial glycogen content [58]. The investigation of blood plasma of rheumatic heart disease of patients by an untargeted LC-MS-based approach indicated an increased level of orotate. The increased level of orotate can be an indicator of a protective mechanism in patients with rheumatic heart disease [59]. On the other hand, the study of Williams et al. (1991) showed that administration of the metabolite orotate increases the pyrimidine nucleotide content in heart tissue [60].

Creatinine is an anhydride form of creatine and is produced in the muscles from creatine phosphate [61,62]. It is removed by the kidneys through glomerular filtration, but also by proximal tubular secretion, and it serves as a marker of kidney function [61,62]. Recent results confirm that renal impairment is strongly associated with outcomes in heart failure patients with systolic dysfunction [63,64]. A previous study indicated that renal insufficiency is common in patients with heart failure, which is accompanied with elevated serum creatinine [65]. Our research showed an elevated level of creatinine in the plasma of dogs with idiopathic dilated cardiomyopathy identified by untargeted LC-MS metabolomics analysis. Nevertheless, the research of Bagheri et al. (2019) demonstrated that patients with coronary artery disease compared with the controls had increased levels of serum urea and creatinine [62]. An increased level of creatinine was recorded during decongestion in patients with acute decompensated heart failure (ADHF). The reasons behind the elevated level of serum creatinine in some patients is not clear. Some explanation for the elevation of serum creatinine is haemoconcentration that occurs during diuretics in ADHF patients or a state of relative hypovolemia in which decreasing left ventricular end-diastolic pressure decreases renal perfusion to a pathological point [66].

The results from statistical analysis confirm that none of the detected metabolites in our study were identified and significantly changed with more than one platform. Furthermore, in the Venn diagram, we can see that 26 identified metabolites are overlapping for the more than one above platforms, confirming the consistency of the above approaches.

The strengths of this research were the use of two complementary metabolomics approaches: untargeted and targeted, and three different analyses: analyses with liquid and gas chromatography coupled with mass spectrometry and flow-injection analysis. The study’s limitation was the small sample size. However, to the authors’ knowledge, this is the first report of a metabolomics-based study for the investigation of metabolite profile and possible changes in the plasma of dogs with idiopathic dilated cardiomyopathy with the highest number of samples.

## 4. Materials and Methods

### 4.1. Experimental Design 

This study was approved by the Committee on Ethics of the University of Zagreb, Faculty of Veterinary Medicine (Permit Number: 640-01/18-17/63; 251-61-20/165-18-01). Two groups of animals were enrolled in the metabolomics study: group 1 consisted of 12 clinically healthy dogs and was used as a control group, and group 2 consisted of 8 dogs diagnosed with iDCM (Table S5A–C). Healthy dogs were admitted to the Clinic for Internal Diseases, Faculty of Veterinary Medicine, University of Zagreb, Croatia. In the control group were dogs with normal cardiac function, of various breeds, and of both sexes (aged from 5 to 12.5 years, 6 males and 6 females). Dogs diagnosed with iDCM were admitted to the Small Animals Clinic, Department of Clinical Veterinary Science, Vetsuisse Faculty, University of Bern, Switzerland. In this group, dogs were of either sex (4 males and 4 females), aged between 3 and 7 years. All dogs included in this study underwent physical examination: blood was drawn for laboratory analysis (complete blood count and biochemical analyses), and cardiac evaluation occurred. Cardiac evaluation included echocardiographic (ECHO) and electrocardiographic (ECG) examination, which were performed in unsedated dogs. The assessment of cardiac rhythm was performed using 1 min 6 lead ECG Aspel AsCard Mr. Silver (Aspel SA, Zabierzów, Poland) and cardiac function by ECHOEsaote MyLab40 Vet machine and a 5 MHz phased array transducer (Davis Medical Electronics Inc., Vista, CA, USA) in a standard manner [67]. All dogs in the disease group were classified according to the International Small Animal Cardiac Health Council (ISACHC) classification system [68].

The diagnosis of IDCM was made based on the finding of enlarged left ventricular M-mode systolic (LVIDs) and diastolic (LVIDd) dimensions defined according to weight-adjusted values or breed-specific values [69,70] and left ventricular M-mode fractional shortening of <20%, both of which represent major criteria for diagnosis of iDCM [71]. The investigation had several exclusion criteria including evidence of any other disease than iDCM based on history, clinical examination, laboratory results, or imaging. The cardiac evaluation of dogs with iDCM included physical examination, thoracic radiographs evaluated by a board-certified radiologist, a 1 min 6 lead ECG AT 101 (SchillerAG, Baar, Switzerland) ), and transthoracic echocardiography performed by aboard-certified cardiologist (AK). Echocardiography was performed using an Aloka ProSound Alpha 5SV machine (Aloka Co., Ltd., Tokio, Japan) and a 5 MHz sector transducer in unsedated dogs and in a standard manner [67]. Dogs with iDCM were classified according to the International Small Animal Cardiac Health Council (ISACHC) classification system [68]. All procedures were conducted in accordance with EU Directive 2010/63/EU for animal experiments, as well as subject to informed owner consent.

### 4.2. Blood Sample Preparation and Analysis

The blood samples were collected from the cephalic vein on the day of admission from all the dogs divided into two groups: 12 clinically healthy dogs (used as controls) and 8 dogs diagnosed with iDCM. The samples were placed in tubes with ethylenediaminetetraacetic acid (EDTA) for haematological and metabolomics analysis and in tubes with no anticoagulant for biochemical analysis. Routine haematology analysis and haematological data of complete blood count were obtained using an automatic haematology analyser Horiba ABX (Diagnostics, Montpellier, France), while biochemical profiles were obtained according to standard methods using an automated biochemistry analyser Olympus AU 600 (Olympus Diagnostica GMBH, Hamburg, Germany). Blood plasma samples were prepared by centrifugation of EDTA tubes at 1600× *g* for 10 min and stored at −80 °C until used for analysis of metabolites. Serum aliquots were prepared by centrifugation of tubes at 1600× *g* for 10 min and used for biochemical analysis. All samples were collected during a one-year period and thawed just once upon completion of collection, immediately before any analysis. Serum and plasma of dogs with iDCM were collected at the time of initial diagnosis and before any treatment.

### 4.3. Untargeted Metabolomics Approach 

#### 4.3.1. Sample Preparation

Metabolites were extracted using extraction solvent consisting of a chloroform/methanol/water (1:3:1, *v*/*v*/*v*) mixture (chloroform, methanol (Honeywell, Charlotte, NC, USA), water (Merck, Darmstadt, Germany)). In short, a total of 25 µL of each serum was subjected to a total of 1000 µL of extraction solvent on a cooled (4 °C) vortex mixer for 5 min. The pooled sample was mixed with a volume of 10 µL of each sample (control and disease sample) and subjected to extraction with extraction solvent. Matrix blank samples contained extraction solvent. All assayed samples (serum samples, pooled samples, matrix blank) were then centrifuged at 13,000× *g* for 5 min at 4 °C. The supernatant (200 µL) was stored at −80 °C until used for UHPLC-MS/MS metabolomics analysis.

#### 4.3.2. Metabolite Analysis

Metabolite extracts were separated on a Dionex UltiMate 3000 UHPLC system (Thermo Fisher Scientific, Germering, Germany) using hydrophilic interaction liquid chromatography (HILIC) and analysed on a Thermo Orbitrap Q Exactive Plus (Thermo Fisher Scientific; Bremen, Germany). The extracts were loaded on a ZIC-pHILIC column (150 mm × 4.6 mm, 5 μm column, Merck Sequant, Darmstadt, Germany) at a column temperature of 30 °C, which was maintained constantly during the analysis. Mobile phase A was 20 mM ammonium carbonate (Honeywell, Charlotte, NC, USA) in water (Merck, Darmstadt, Germany)). Mobile phase B was 100% acetonitrile (Honeywell, Charlotte, NC, USA). Metabolites were eluted using a linear gradient from 80% of mobile phase B to 5% with a flow rate of 0.3 mL/min. The injection volume was a volume of 10 μL in every run, and samples were maintained in the autosampler at 5 °C prior to injection. A Thermo Orbitrap Q Exactive Plus instrument (Thermo Fisher Scientific; Bremen, Germany) was operated in polarity switching mode with electrospray ionisation at a mass resolution of 70,000 FWHM and the full scan of *m*/*z* range 70–1050. The MS setting was acquired for positive and negative polarities as follows: source voltage of +3.8 kV for positive and −3.8 kV for negative modes, sheath gas 40 (arbitrary units), auxiliary gas of 5 (arbitrary units), and capillary temperature of 320 °C. Metabolites were identified using a standard sample mix (kindly provided by Glasgow Polyomics, Glasgow, UK) of 148 reference compounds. Quality control samples were extracts obtained from foetal bovine serum and beer (kindly provided by Glasgow Polyomics, Glasgow, UK) and used in the analysis to check signal reproducibility and the quality of chromatography.

#### 4.3.3. Data Processing

The results of metabolomics analysis were processed with the MSconvert tool for conversion of data (ProteoWizard Software Version 3, San Diego, CA, USA) and the Polyomics integrated Metabolomics Pipeline (PiMP) available at http://polyomics.mvls.gla.ac.uk (accessed on 12 June 2023) for metabolite identification [72]. The analysis components in PiMP (http://polyomics.mvls.gla.ac.uk, accessed on 12 June 2023) are implemented as an R pipeline based around XCMS [73] for the feature detection and mzMatch.R [74] for common metabolomics data pre-processing tasks (e.g., alignment, batch correction, and identification). All these components are gathered in a Docker container for easy deployment, both locally and on a shared server. The peaks for each sample were called and retention time corrected using the Obiwarp algorithm [75]. Filtering was carried out on the basis of noise, number of values present, etc. The raw LC-MS data obtained from each sample were converted from the Thermo Scientific “RAW” file format to an open-source “mzXML” file format, centroided, split into positive and negative polarities using ProteoWizard software Version 3 (San Diego, CA, USA) [76]. The metabolite was identified in PiMP software available at http://polyomics.mvls.gla.ac.uk (accessed on 15 July 2023), according to the metabolomics standards initiative (MSI) guidelines, based on mass and retention times of detected peaks with authentic standard mix while features were annotated based on am accurate mass of standards in a metabolite libraries search (e.g., The Human Metabolome Database, HMDB available at https://hmdb.ca/ (accessed on 9 August 2023) and/or Kyoto Encyclopaedia of Genes and Genomes, KEGG available at https://www.genome.jp/kegg/ (accessed on 9 August 2023) integrated within PiMP software available at http://polyomics.mvls.gla.ac.uk (accessed on 15 July 2023). Information on which metabolites were identified using retention time and *m*/*z* (Level 1 MSI) and which features were annotated using *m*/*z* only (Level 3 MSI) are given in Table S6. The reported compounds using PiMP software available at http://polyomics.mvls.gla.ac.uk (accessed on 15 July 2023) in the Tables S2 and S6, marked as “Annotation”, represent tentative identifications. These identifications exclude correct annotations of fatty acyl constituents, positional isomers, double bond positions, and configurations. These annotations need to be further confirmed using authentic standards. This confirmation process involves comparing retention time, accurate mass (MS1), and MS/MS spectrum.

### 4.4. Targeted Metabolomics Approach

Metabolite extracts were performed using the Absolute IDQ p400 kit (Biocrates Life Science AG, Innsbruck, Austria), a commercially available kit for targeted metabolomics analysis of up to 408 metabolites distributed into 11 metabolite classes. This kit allowed for the detection and quantification of different class of metabolites combining liquid chromatography–mass spectrometry (LC-MS/MS) and flow-injection analysis–mass spectrometry (FIA-MS/MS). The LC-MS/MS analysis quantified amino acids and biogenic amines while the FIA-MS/MS was used to quantify acylcarnitines, glycerophospholipids, glycerides, hexoses, cholesteryl esters, and sphingolipids.

#### 4.4.1. Sample Preparation

Sample preparations for targeted metabolomics analysis were performed based on manufacturing protocols provided with the kit. The metabolite extracts were prepared on the specific 96-well plate system in three steps: protein removal, internal standard normalisation, and derivatisation. In short, a volume of 10 μL of plasma samples, calibration standards, zero standards (phosphate-buffered saline (BDH PROLABO, Lutterworth, UK)), and quality control samples (QC 1–3, the QC2 samples was injected in five replicates) were added to the centre of the filter on the 96-well kit plate containing the internal standard mix. One blank sample was also added on the plate. The samples were then dried for 30 min using a vacuum manifold (Thermo Scientific, Waltham, MA, USA) and derivatised with 50 μL of 5% derivatisation solution of phenylisothiocyanate (PITC) (Sigma-Aldrich, St. Louis, MO, USA) in a mixture of water/ethanol/pyridine at ratio of 1:1:1 (water (Merck, Darmstadt, Germany), ethanol (Honeywell, Charlotte, NC, USA), pyridine (BDH PROLABO, Lutterworth, UK)). Then, the 96-well kit plate was incubated for 20 min at room temperature and was dried for 60 min using vacuum manifold (Thermo Scientific, Waltham, MA, USA). The metabolite extraction was produced by the addition of 300 μL of 5 mM ammonium acetate (Sigma-Aldrich, St. Louis, MO, USA) in methanol (Honeywell, Charlotte, NC, USA) and was shaken at 450 rpm for 30 min. The metabolite extracts were collected using a vacuum manifold (Thermo Scientific, Waltham, MA, USA) for 2 min into a capture plate for a FIA-MS/MS analysis. A total of 250 μL of FIA mobile (mix of 290 mL MeOH and a 10 mL ampule Biocrates FIA mobile phase additive, provided with the kit) was added directly to each well of the original capture plate, while a total of 150 μL from the capture plate was transferred to another plate and diluted with 150 μL LC-MS-grade water for LC-MS/MS analysis.

#### 4.4.2. Metabolite Analysis

Plasma samples were analysed on a Dionex Ultimate 3000 UHPLC system (Thermo Fisher Scientific, Germering, Germany) coupled to a Q Exactive Plus hybrid quadrupole-Orbitrap mass spectrometer (Thermo Fisher Scientific, Bremen, Germany), using a Thermo p400 HR UHPLC column provided with the kit (available from Biocrates), and the column temperature was maintained at 50 °C. Mobile phase A was 0.2% formic acid (Sigma-Aldrich, St. Louis, MO, USA) in H_2_O (Merck, Darmstadt, Germany), and mobile phase B was 0.2% formic acid (Sigma-Aldrich, St. Louis, MO, USA) in acetonitrile (Honeywell, Charlotte, NC, USA). The injection volume was 5 μL. The total run time was 5.81 min, and the gradient change of 0 to 95% of mobile phase B over 4 min at flow rate was 0.8 mL/min. For the FIA-MS/MS analysis, metabolites were eluted using FIA mobile phase at flow rate 0.05 mL/min for the first 1.6 min, and then the flow rate increased to 0.2 mL/min for 1.2 min and then decreased back to 0.05 mL/min for the rest of the program. The instrument analysis was performed in positive and negative polarities for LC-MS/MS and FIA-MS/MS according to the instructions from Biocrates (Biocrates Life Science AG, Innsbruck, Austria). Briefly, the Orbitrap mass spectrometer was operated in full-scan acquisition mode using electrospray ionisation at a mass resolution of 70,000 FWHM and a full scan of the *m*/*z* range of 100 to 800 for LC-MS/MS analysis, and 100 to 1000 for FIA-MS/MS. The microscans were 1, AGC target of 1 × 10^6^, and maximum injection time (IT) of 250 ms. The HESI source settings were acquired with a source voltage of 3.0 kV for LC-MS/MS analysis and 2.50 kV for FIA-MS/MS with a capillary temperature of 300 °C. In the LC-MS/MS metabolomics analysis, the sheath gas was 60 (arbitrary units), auxiliary gas was 30 (arbitrary units), and S-lens RF level was 60 for LC1 and 90 for LC2 analysis with an aux gas heater temperature of 550 °C. For the FIA-MS/MS metabolomics analysis, the sheath gas was 15 (arbitrary units), auxiliary gas was 5 (arbitrary units), and S-lens RF level was 60 with an aux gas heater temperature of 120 °C.

#### 4.4.3. Data Processing

The data analysis was performed according to the manufacturer’s guidelines using the Biocrates MetIDQ software Version Boron (Biocrates Life Science AG, Innsbruck, Austria). The quantification of the LC-MS metabolites was processed via XCalibur Quan 4.1 software (Thermo Fisher Scientific, Waltham, MA, USA) based on a seven-point calibration curve and isotope labelled internal standards for most analytes. The FIA-MS/MS analysis used a single-point calibrator with representative internal standards. Blank samples (phosphate-buffered saline) were used for the calculation of the limits of detection (LOD). If the compounds were quantified with restriction, then the calibration curves had expected coefficients of determination (R^2^) < 0.99 according to the manufacturer guidelines. On the other hand, when specific standards were not commercially available and verification of the accuracy was not possible by the manufacturer, then in terms of quantification, the measuring was “semi-quantitatively”.

### 4.5. GC-MS-Based Metabolomics Analysis

#### 4.5.1. Sample Preparation

The solution for metabolite extraction was prepared by mixing water/methanol/chloroform at a ratio of 1:2.5:1 (water (Merck, Darmstadt, Germany), methanol, and chloroform (Honeywell, Charlotte, NC, USA)). A total of 25 µL of each plasma sample was extracted by mixing with a volume of 250 µL of extraction solution, followed by incubation for 30 min at 37 °C and centrifugation at 16,000× *g* for 5 min at 4 °C. A volume of 225 µL of the obtained extracts was subjected to 200 µL of distilled water, shaken at 1200 rpm for 30 min at 37 °C, and centrifuged at 16,000× *g* for 5 min at 4 °C. The supernatant (225 µL) was evaporated to dryness using a speedvac concentrator (Thermo Fisher Scientific, Waltham, MA, USA) for 4 h. A dried aliquot was reconstituted with 40 µL methoxyamine hydrochloride (20 mg/mL) (Sigma-Aldrich, St. Louis, MO, USA) in pyridine (BDH PROLABO, Lutterworth, UK). The resultant solution was shaken at 1200 rpm for 90 min at 30 °C. Subsequently, a volume of 20 µL of *N*-methyl-*N*-trimethylsilytrifluoroacetamide (MSTFA) (Sigma-Aldrich, St. Louis, MO, USA) was added in the resultant solution, followed by incubation at 1200 rpm for 30 min at 37 °C and centrifugation at 16,000× *g* for 5 min at 20 °C. The prepared extracts were stored at −80 °C until GC-MS analysis.

#### 4.5.2. Metabolite Analysis

The instrumental analysis was carried out on a Shimadzu single quadrupole GCMS-QP2010 gas chromatograph–mass spectrometer (Shimadzu, Kyoto, Japan) with electron ionisation as the ion source. Metabolites were loaded on a 30 m × 0.25 mm × 0.25 μm BPX-5 capillary column (SGE, Austin, TX, USA), and a total of 1 μL of each derivatised sample was split injected with ratio of 1:80. The injector port temperature was held at 250 °C, and the carrier gas helium was used at a constant flow rate through the column of 1 mL/min. The total run time was 60 min for each plasma sample. The temperature was programmed at 60 °C for 2 min, then increased to 330 °C at 15 °C/minute and maintained for 10 min. MS ion source temperature was 200 °C, and the interface temperature was 280 °C. Mass range was 45–600 *m*/*z* with a scan time 1 s.

#### 4.5.3. Data Processing

The GC-MS metabolomics data were analysed using the Shimadzu GCMSsolution software Version 2.53 (Shimadzu, Kyoto, Japan). The retention time correction of detected peaks was performed by adjusting it with the retention time of a standard alkane series mixture (C_10_ to C_40_) in the automatic adjustment of retention time (AART) function of the Shimadzu GCMSsolution software Version 2.53 (Shimadzu, Kyoto, Japan). Low-molecular-weight metabolites were identified by a commercially available GC/MS Metabolite Mass Spectral Database (Shimadzu Co., Kyoto, Japan). The GC/MS MS Metabolite Mass Spectral Database contained a mass spectral library (method files that specified the above-described analytical conditions) and the parameters for data analysis of 178 compounds (amino acids, fatty acids, and organic acids). A similarity index was calculated based on retention time and the confirmed ion and fragmentation pattern obtained in the mass spectrum of the low molecular weight metabolites. Peaks with a similarity index of less than 80 were processed as unknown molecules, while peaks with a similarity index more than 80 were identified metabolites. The peak height of 2-isopropylmalic acid was used as an internal standard to perform the semi-quantitative assessment (the peak height of each quantified ion was calculated and normalised using the peak height of internal standard).

### 4.6. Statistical Analyses

Online available software MetaboAnalyst v.4.0 (http://www.metaboanalyst.ca, accessed on 20 July 2023) was used for statistical analyses [77]. Metabolite contents were compared using Student’s *t*-test for both group of dogs with FDR correction systematically applied across all *t*-tests. Metabolites with a *p*-value < 0.05 were considered statistically significant. For the untargeted metabolomics, all statistical analyses were performed on the combined positive and negative ion data sets, exported as a peak intensity table from PiMP (http://polyomics.mvls.gla.ac.uk (accessed on 25 July 2023)). The untargeted data were normalised by median, log transformed to improve normality, and mean centred. In the targeted data, features with 50% missing values were removed and replace by LoDs (1/5 of the minimum positive value of each variable), normalised by sum, log transformed, and Pareto scaled, while the GC-MS data were normalised by sum, log transformed, and Pareto scaled prior to partial least squares–discriminant analysis (PLS-DA) and the variable importance on projection (VIP). A supervised PLS-DA classification method was used to identify the important metabolites, while a variable importance in the projection (VIP) plot was ranked the metabolites based on their importance in discriminating the dogs with iDCM from healthy dogs. Metabolites with the highest VIP values are the most powerful group discriminators, and therefore metabolites with VIP values >1 are significant and metabolites with VIP values > 2 are highly significant.

## 5. Conclusions

We observed a pattern of significantly altered metabolites that has contributed that the metabolomics profiles of dogs with iDCM distinguish significantly from healthy dogs. The study combined untargeted and targeted metabolomics approaches by using liquid and gas chromatography coupled with mass spectrometry and flow-injection analysis. The combination of these analytical platforms resulted in the identification of 272 metabolites. Our findings demonstrated that iDCM was associated with changes in the concentration of amino acids, biogenic amines, acylcarnitine, triglycerides and diglycerides, sphingomyelins, and organic acids. The results of this study provide a global overview of the metabolome in dogs with iDCM and provided a new perspective into the metabolic abnormalities identified herein. Additionally, our results highlight new targets for further investigation.

## Figures and Tables

**Table 1:** List of identified and significantly changed metabolites in plasma of dogs with iDCM versus healthy dogs performed by the untargeted LC-MS approach.

Metabolites	Peak ID	Mass	RT(s)	*p*-Value (FDR)	Log_2_ (FC)
Cystine	1021	241.0309	730.41	0.043	−0.84
4-Hydroxyproline	180	132.0656	684.95	0.045	−0.69
Creatinine	4	114.0662	525.25	0.030	0.45
3-Hydroxybutanoate	2002	103.0403	514.83	0.030	0.71
Orotate	2559	155.0103	555.73	0.001	0.94
Lactate	1367	89.0246	528.32	0.025	1.00
Carnitine	3	162.1124	636.28	0.023	1.70
*cis*-Aconitate	2003	173.0097	800.83	0.0065	1.72
*O*-Acetyl-L-carnitine	21	204.123	553.67	0.010	2.20
3-Metylhistidine	27	170.0924	616.4	0.034	2.37

## Data Availability

The mass spectrometry metabolomics raw data are available on request to the correspondence author. All results are presented within the manuscript and/or Supplementary Materials.

## Supplementary Materials

The supporting information can be downloaded at: https://www.mdpi.com/article/10.3390/ijms242015182/s1.
